# Prevalence of Congenital Heart Defects in Pregnancies Conceived by Assisted Reproductive Technology: A Cohort Study

**DOI:** 10.3390/jcm10225363

**Published:** 2021-11-18

**Authors:** Alessandro Galdini, Vlasta M. E. Fesslova, Gerarda Gaeta, Massimo Candiani, Mirko Pozzoni, Carmelina Chiarello, Paolo Ivo Cavoretto

**Affiliations:** 1 Gynecology and Obstetrics Department, IRCCS San Raffaele Hospital, University Vita-Salute, Via Olgettina 60, 20132 Milan, Italy; galdini.alessandro@hsr.it (A.G.); gaeta.gerarda@hsr.it (G.G.); candiani.massimo@hsr.it (M.C.); pozzoni.mirko@hsr.it (M.P.); cavoretto.paolo@hsr.it (P.I.C.); 2 Center of Fetal Cardiology, IRCCS Policlinico San Donato, Via Morandi 30, 20097 Milan, Italy; 3 Department of Congenital Cardiac Surgery, IRCCS Policlinico San Donato, Via Morandi 30, 20097 Milan, Italy; Carmelina.Chiarello@grupposandonato.it

**Keywords:** in vitro fertilization, IVF, ICSI, intracytoplasmic sperm injection, assisted reproductive technology, ART, congenital heart defects, fetus, pregnancy, fetal ultrasound, fetal echocardiography

## Abstract

Background and aim of the study: Pregnancies obtained by assisted reproductive technology (ART) are associated with an increased risk of complications and congenital anomalies, particularly congenital heart defects (CHDs). Therefore, our aim is to evaluate, retrospectively, the prevalence of CHD in ART pregnancies in our two centers and analyze their characteristics and outcomes. Methods: Observational study including fetuses conceived by ART referred between June 2011 and September 2020 and undergoing a fetal cardiac ultrasound scan. Cases with genetic, chromosomal abnormalities or extracardiac malformations were excluded. Population included 1511 pregnancies, which consisted of 269 twins and 1242 singletons, 547 IVF (in vitro fertilization), 773 ICSI (intracytoplasmic sperm injection) and 191 oocyte donations (OD). Results: CHDs were found in 29 fetuses, with an overall prevalence of 1.92% (29/1511), 1.85% (23/1242) in singletons and 2.23% in twins (6/269). Thirteen were IVF, eight ICSI and eight OD cases, with a greater risk of CHD after IVF and OD (IVF: 13/29 (44.8%)—one twin; ICSI: 8/29 (27.6%)—three twins); 22 had major and 7 minor defects. Two pregnancies with a hypoplastic left heart were terminated; the majority of live-born cases needed surgery. Three babies died (two post-surgery, one had a late death). Conclusions: Our data show an increased prevalence of CHD after ART with a heterogeneous spectrum of diagnoses, mainly major defects.

## 1. Introduction

The use of assisted reproductive technology (ART) has increased during recent years, especially high-technology techniques such as in vitro fertilization (IVF), intracytoplasmic sperm injection (ICSI) and oocyte donation (OD). In the United States, approximately 1.9 percent of all births result from ART [1], whereas in Europe, in several countries, they are reported rates of up to 2–4%. In Italy, the rate of births resulting from ART tripled during the last fifteen years, nowadays reaching 2.4 percent of all births. ART is an essential opportunity enabling reproduction in many sterile or infertile couples. However, it was ascertained that conception by IVF/ICSI is associated with increased risks of several obstetric and perinatal complications such as multiple gestations, a preterm delivery, low birth weight and congenital anomalies [2,3,4,5,6]. Several reviews and meta-analyses have shown that the risk of congenital anomalies is potentially increased by approximately one-third with respect to spontaneous pregnancies [7,8,9,10,11,12,13]. All organs and systems appear to be affected, with particular interest in congenital heart defects (CHD) [14,15]. In the general population, the risk of CHD is about 0.76% in live births and 0.61% when excluding underlying genetic diseases [10]; however, some studies report an incidence of up to 12–14/1000 live births [16,17]. Recently, our group published a meta-analysis showing that the risk of CHD in IVF/ICSI pregnancies is significantly higher, by about 50%, as compared with pregnancies conceived spontaneously; in particular, we found a prevalence of congenital heart defects at birth of 13 per 1000 births in IVF/ICSI pregnancies [18].

However, there is still no consensus upon the clinical utility of performing fetal echocardiography in ART pregnancies. Only the American Heart Association (AHA) guidelines include IVF/ICSI conception among maternal indications for fetal echocardiography with a recommendation class/level of evidence of IIa/A estimating an absolute risk of 1.1–3.3% among live births [19]. Furthermore, data regarding the specific subtype of CHD in the literature are discordant. In recent years, we have observed an increasing number of ART pregnancies referred for a detailed ultrasound fetal cardiac evaluation in our centers.

## 2. Objective

The aim of our study was to analyze the prevalence of fetuses with CHD in ART pregnancies in our two centers, to define their characteristics and outcomes and to compare our data with those obtained from the literature.

## 3. Materials and Methods

### 3.1. Study Design and Patients Characteristics

This was a observational retrospective cohort study, including fetuses conceived after ART and referred to two Italian centers of prenatal diagnosis (IRCCS Ospedale San Raffaele, Milano, and IRCCS Policlinico San Donato Milanese, San Donato, Milano), between June 2011 and June 2020 for ultrasound evaluation of the fetal heart.

Cases were included in the study if they met the following criteria: (a) the pregnancy was conceived by high-technology ART (with IVF, ICSI or OD) with or without the use of oocyte or embryonic cryopreservation; (b) fetal cardiac scans were performed at our institutions during the second or third trimester of gestation and ultrasound report was available; (c) availability of neonatal data regarding general status and detailed pediatric cardiological follow-up in cases with congenital heart defects. Fetuses were excluded in cases of genetic or chromosomal abnormalities (suspected or confirmed), syndromes and cases with associated major extracardiac malformation. No cases of spontaneous abortions in ART pregnancies with a diagnosed congenital heart disease (CHD) occurred and, therefore, such cases were not included in the study.

Retrospectively, ultrasound reports and pregnancy characteristics were reviewed in order to extract data on maternal age, gestational age, number of fetuses and type of ART: homologous (HO) or heterologous conceptions (OD). In case of homologous ART, we distinguished the use of ICSI or IVF.

The cases were divided into two study groups: (1) pregnancies with a fetus with a suspected cardiac anomaly—congenital heart defect (CHD)—at the prenatal exam and confirmed after birth via imaging techniques (ultrasound, CT-scan, MR) or during surgical procedure (group named CHD-ART); (2) pregnancies with a normal cardiac anatomy at the prenatal exam with no neonatal problems reported (group non-CHD-ART). The types of fetal CHD were specified and classified in homogeneous groups of anomalies and further distinguished in major and minor defects.

### 3.2. Ultrasound Methods

We specified whether a standard ultrasound cardiac scan or a specialist fetal echocardiography was performed. If the woman performed both, only fetal echocardiography was considered.

All ultrasound cardiac evaluations were performed by a limited group of senior operators with extensive experience and expertise in this field, including fetal cardiologist and subspecialist in fetal medicine with competence in fetal cardiac scanning.

Standard ultrasound cardiac evaluation was performed by obstetricians subspecialists in fetal medicine and with a certification of advanced fetal cardiac assessment granted by the Fetal Medicine Foundation, whereas fetal echocardiography was performed by a specialist in fetal cardiology and included additional views according to the case and the maximum level of expertise.

All cardiac ultrasound scans were performed according to ISUOG guidelines and included 4-chamber view, outflow tracts, three vessels and tracheal view, aortic and ductal arches views, the inferior and superior vena cava view [20,21]. Color Doppler was applied on these views and M-mode and spectral Doppler/Color Doppler.

The following ultrasound machines were used during the study period: Voluson E8 or E10, (GE Healthcare, Zipf, Austria) and Aloka ProSound F75 Premier (Hitachi Aloka Medical Ltd., Mure Mitaka, Japan), equipped with multifrequency convex transducers.

Neonatal echocardiography was performed according to the current criteria in cases with a prenatal diagnosis of congenital heart defect or in suspicious ones.

Neonatal outcome was checked directly retrieving neonatal data from the neonatologists or documents from the parents, both in the cases with CHD and in those with a normal cardiac anatomy.

### 3.3. Statistical Analysis

The prevalence of CHDs in our population was calculated firstly as follows:

$$\mathrm{Prevalence}\;\mathrm{of}\;\mathrm{CHD}\;=\;\frac{\mathrm{Total}\;\mathrm{number}\;\mathrm{of}\;\mathrm{CHDs}\;\mathrm{diagnosed}\;\mathrm{in}\;\mathrm{the}\;\mathrm{study}\;\mathrm{period}\;\mathrm{in}\;\mathrm{ART}\;\mathrm{pregnancies}}{\mathrm{Total}\;\mathrm{number}\;\mathrm{of}\;\mathrm{ART}\;\mathrm{pregnancies}\;\mathrm{undergoing}\;\mathrm{fetal}\;\mathrm{cardiac}\;\mathrm{scanning}\;\mathrm{in}\;\mathrm{the}\;\mathrm{study}\;\mathrm{period}}$$

Comparison of continuous and categorical variables between the two study groups (CHD ART and non-CHD ART) was performed with Student’s *t*-test or χ^2^ test, as appropriate. With the same method, a subanalysis limited to singleton pregnancies was performed. Subanalysis was not performed in twin pregnancies, due to a limited number of cases. *p*-value < 0.05 was considered significant. Data were analyzed with SPSS software (IBM SPSS Statistics for Windows, version 20; IBM Corp., Armonk, NY, USA).

## 4. Results

**Figure 1:**
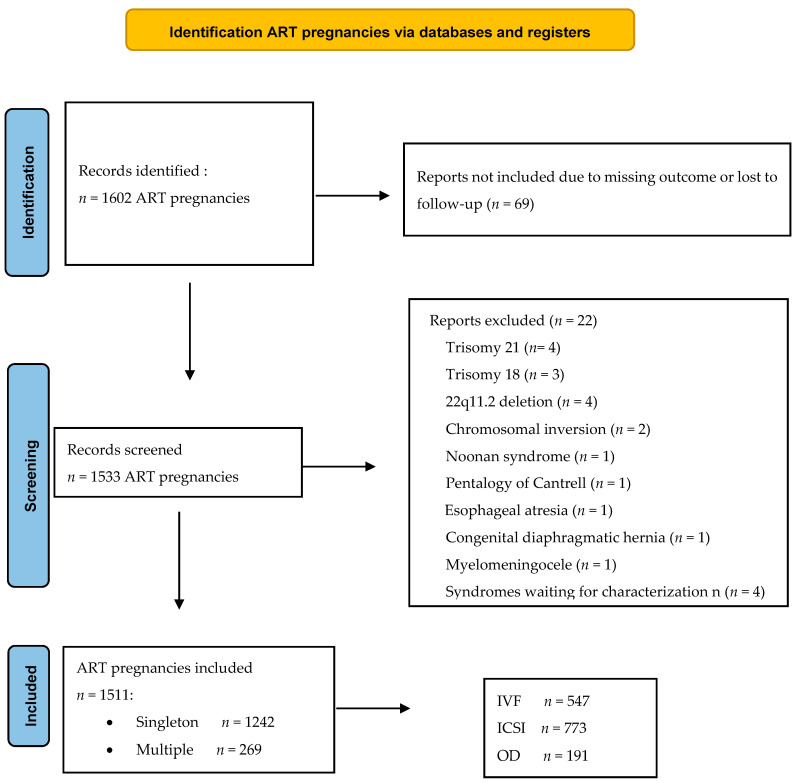
Flow chart of ART cases. ART—assisted reproductive technology (ART); IVF—in vitro fertilization; ICSI—intracytoplasmic sperm injection; OD—oocyte donation.

Over the 9-year study period, we evaluated 1602 ART pregnancies, of which 1533 had available outcome data and were, therefore, considered for analysis (Figure 1).

Twenty-two cases were excluded from the study according to the exclusion criteria: trisomy 21 (*n* = 4), trisomy 18 (*n* = 3), 22q11.2 deletion (*n* = 4), chromosomal inversion (*n* = 2), Noonan syndrome (*n* = 1), Pentalogy of Cantrell (*n* = 1), esophageal atresia (*n* = 1), congenital diaphragmatic hernia (*n* = 1), myelomeningocele (*n* = 1) and syndromes waiting for characterization (*n* = 4). After the selection of cases, 1511 pregnancies were included in the analysis (Figure 1). The mean maternal age was 37.7 years (range 22–55 years), median gestational age at the ultrasound assessment was 23 gestational weeks (range 16–37). A total of 1124 (74.4%) underwent a standard heart evaluation by obstetrician subspecialists in fetal medicine, and 387 underwent a specialist fetal echocardiography with a fetal cardiologist (25.6%). There were 269 twin pregnancies (17.8%), of which 39 were monochorionic and 5 triplets. Pregnancies conceived after homologous ART were 1320 (87.4%; 773 ICSI and 547 IVF) and after heterologous ART (OD—oocyte or embryo donation) were 191 (12.6%). Table 1 shows the characteristics of the study population and specifies the pregnancies with respect to the type of ART.

CHDs were found in 28 pregnancies and 29 fetuses (6 fetuses from 5 twin pregnancies and 24 singleton pregnancies); the prevalence of CHDs in the study population was 1.85% (28/1511) considering the number of affected pregnancies and 1.92% (29/1511) considering the number of affected fetuses. The prevalence in all ART singleton pregnancies was 1.85% (23/1242) and in twin pregnancies was 1.86% (5/269; there were six fetuses with CHDs from five pregnancies with a rate of CHD in twins of 2.23%; 6/269 fetuses).

Table 2 shows the comparison of variables and pregnancy characteristics between the two study groups, specifically, for singleton and multiple pregnancies.

Considering the type of ART, 21 CHD cases were found in the group of homologous ART and 8 in the group of heterologous ART (OD). Most of the CHDs were found in the IVF group as compared to the ICSI group (IVF: 13/29; 44.8%—one twin; ICSI: 8/29; 27.6%—three twins (Table 3).

### 4.1. Description of the Cardiac Abnormalities

The majority of the CHDs were major defects (22/29); seven were minor defects (Table 3).

Among the CHDs, we observed more frequently: Tetralogy of Fallot (ToF)/Pulmonary atresia with a ventricular septal defect (VSD) (five cases), hypoplastic left heart syndrome (HLHS) (four cases) and atrioventricular septal defect (AVSD) (three cases). These defects were not associated with a specific type of ART conception, except for the cases of HLHS, all obtained with ICSI. Only three VSDs were found, one muscular and two perimembranous.

### 4.2. Pregnancy and Neonatal Outcomes

Of the 22 major CHDs, in two cases with HLHS, a termination of the pregnancy (TOP) was performed. There were no cases of intrauterine fetal deaths. In four pregnancies (of which three were twin pregnancies), a preterm birth before 37 gestation weeks occurred: two were spontaneous at 34 and 36 weeks and the other two were iatrogenic at 36 weeks for the development of preeclampsia or hypertensive disorders with fetal growth restriction in one. The remaining fetuses were born at term, by cesarean section in 65% of cases (17/25), performed according to obstetrics indications.

In all live birth babies, a postnatal confirmation of the prenatal diagnosis of a CHD was performed by means of a specialist echocardiography. Fifteen out of twenty (75%) live born babies with major CHDs underwent a surgical repair, in the neonatal period or within the first year of life, with a good outcome in twelve. In two cases with HLHS, a neonatal death occurred after the first and second stage Norwood operation, and another case with ToF/pulmonary atresia+ VSD died late at 11 months, after a previous postnatal systemic-to-pulmonary shunt operation.

## 5. Discussion

### 5.1. Prevalence of Congenital Heart Disease in ART Pregnancies

Our study showed a prevalence of CHDs in ART pregnancies of 1.92% (1.85% in singletons and 2.23% in twins). These values were increased with respect to the data of spontaneous pregnancies in normal populations [10]. In the general population, the EUROCAT registry reported a CHD prevalence of about 0.76% in live births and 0.61% when excluding underlying genetic diseases [16].

In contrast to some other reports [7,22], we found a lesser prevalence of CHDs in the cases obtained by ICSI, in which there might have been a more supposed relevant interference with the embryonal evolution. Additionally, we found a higher prevalence of CHDs after heterologous conception, and the significance of this deserves further investigation.

Other smaller case series of ART pregnancies referred to a single center for a fetal cardiac scan were published in the past and the reported variable prevalence of CHDs, and we believe that these differences might be explained by the divergence of methodological aspects of the study design [23,24,25].

In the study of Bahtiyar et al. [23], the frequency of CHD was 1.1% per pregnancy (8 out of 749 IVF pregnancies), but women examined, with additional referral indications to that of IVF gestation, were excluded from the study. Patil et al. [24] evaluated 264 pregnancies obtained with ART conception and 8/264 pregnancies presented an isolated cardiac anomaly at the fetal echocardiography (rate of 3%); however, these were mostly mild cardiac anomalies (VSDs, ventriculomegaly, ventricular free wall thickening, pericardial effusions, tricuspid regurgitation). The meaning of this result is uncertain due to the lack of postnatal confirmation of the findings. In the case review of Aderibigbe et al. [25], cardiac abnormalities (ventricular septal defects, post-valvular pulmonary artery dilation, right aortic arch, aberrant right subclavian artery) were detected in 8 out of 85 pregnancies conceived with IVF/ICSI (rate of 7%); however, the generalizability of this study was limited by some limitations, including the small sample size, lack of a postnatal confirmation of CHDs in all cases and no surgical interventions described in the early newborn period.

Previously, a meta-analysis from our group showed major evidence of an increased risk of CHDs in ART pregnancies [18]. The study included data on neonates, infants, intrauterine fetal demises, stillbirths, terminations of pregnancy and excluded cases with chromosomal abnormalities. According to this meta-analysis, children conceived via IVF/ICSI had a 1.3% incidence of CHD (337 out of 25856) as compared to 0.68% in naturally conceived pregnancies (pooled OR, 1.45; 95% CI, 1.20–1.76; *p* = 0.0001; I2 = 44%; *p* = 0.08). Several sensitivity analyses conformed the stability of these results.

The prevalence of CHDs in ART pregnancies of our current cohort study was higher than that reported in spontaneous conceptions and it was also higher than that reported in the IVF/ICSI group of the above-mentioned meta-analysis. One explanation for this increased rate could be that our services of prenatal diagnosis served as referral centers for our region, for a high-risk obstetric population that may have a major possibility of cardiac anomalies.

### 5.2. Twin Pregnancies

In twin pregnancies, our study found a prevalence of CHDs of 2.23% when considering the number of fetuses with CHDs (6/269) and 1.86 when considering the number of pregnancies (5/269). Although the number of twin pregnancies of our study was limited and the study was not designed to address this question, our results did not suggest that the prevalence of CHDs in ART pregnancies is influenced by twinning, as stated by previous reports [26]. However, it was clear that, occasionally, both twins may be affected, as in one of our pregnancies, and that the risk of CHDs of twin pregnancies in ART is still a matter of debate, being importantly related with monochorionicity.

The use of ART has been associated with an increased rate of twin pregnancies [1,27], mainly related to the number of embryos transferred. As a consequence, previous studies suggested that part of the increased risk of CHDs observed in ART pregnancies may be attributed to an increased number of twins, but not to the ART treatment itself. However, in the general population, the risk for all congenital anomalies is increased in twins with respect to the singletons [28,29,30]. On the other hand, Wen et al. [26] published the results of a large cohort study of 507,390 singleton or twin pregnancies, where the prevalence of CHDs was higher in ART pregnancies (*n* = 223; 2.2%) than in non-assisted pregnancies (*n* = 6057; 1.2%). With the use of a mediation analysis, authors stated that twinning contributed up to 87% of the association between ART and CHDs. Previous studies [31,32], regarding the contribution of twinning in the literature, used different strategies to analyze the association of ART, twinning and CHDs, and considered mostly twinning as a confounder, coming to different conclusions. Panagiotopoulou et al. found a higher incidence of CHDs in ART twins independent of chorionicity and other covariates, including the maternal age, parity and gender of the offspring (OR 2.60, 95% CI 1.24–5.45) [31].

### 5.3. Types of CHD

#### 5.3.1. Major CHD

HLHS and conotruncal anomalies (mainly TOF) were the most frequently found CHDs in our study. In our previous meta-analysis [18], HLHS was more frequent in IVF/ICSI pregnancies (1/353; 0.28%) as compared to the spontaneous conception group (5/9526; 0.05%), but this apparently divergent distribution was not statistically significant. Another report [33] did not find an association between HLHS and IVF treatment.

Out of the malformations of the outflow tracts and ventriculoarterial connections, TOF was the most common (five cases), followed by TGA (two cases) and DORV (one case). Published data on this topic are controversial. Previous evidences from our group [18], as well as from others’ [34], did not confirm an association between IVF and TOF; on the other hand, other authors found a link between ART and conotruncal defects [14]. In particular, Tararbit et al. [32,33] found that ART was associated with a 2.4-fold increased risk of TOF, after taking into account the maternal age, occupation, geographic origin, paternal age and year of birth. Especially, ICSI was associated with three-fold higher adjusted odds of TOF. A further adjustment for multiple pregnancies and the exclusion of chromosomal anomalies did not substantially modify these results. Contrarily, the risk of others defects such as TGA and CoA was not increased, leading the above-mentioned authors to speculate a potential implication of neural crest cells in the association between ART and TOF. Developmental origins of CHD and all their subtypes are complex and not fully understood; therefore, more research would be needed on this topic.

#### 5.3.2. Minor CHD

The meta-analysis of our group showed an increased rate of VSDs in IVF/ICSI pregnancies as compared to spontaneous conceptions. [18] Other case series reported similar findings [25,35], particularly Koivurova et al. [35], who reported of 4 VSDs out of 304 IVF pregnancies (1.3%) versus 2 VSDs out of 569 in spontaneous conception (0.4%). In our series, we found only three VSDs, one muscular and two perimembranous. However, we could consider a possible bias of our study that is based on prenatal ultrasound exams, mainly performed during the second trimester of pregnancy, when small isolated VSDs are often difficult to detect. Differently, other studies reported data obtained by birth registries or other postnatal medical records. Postnatal studies probably include a greater proportion of VSDs, which may be missed prenatally. In fact, after birth, it is easier to diagnose VSDs with an ultrasound or the detection of murmurs, even those with a smaller size and minor hemodynamic relevance; it is; therefore, expected to observe a higher prevalence.

### 5.4. Study Strengths and Limitations

Our cohort study included a significant number of ART pregnancies seen in two centers of prenatal diagnosis. In addition, the information gathered from our databases excluded associated genetic or other congenital anomalies. All ultrasound cardiac evaluations were performed by a small group of senior operators with great experience in this field; in particular, V.F. performed essentially all fetal echocardiographies. All cases with prenatal suspected CHDs were confirmed postnatally. Postnatal data were available in all cases. Our collection data based on prenatal assessments had the advantage of including cases of terminations of pregnancy or stillbirths, which may account for severe congenital defects that may be excluded from registry-based postnatal studies. We presented data regarding both singleton and multiple pregnancies. Separate subanalyses for the singleton and twin pregnancies avoided potential confusion related to twinning. Recent work from our group showed increased rates of placental-related obstetric complications, including preeclampsia, preterm birth and a low-birth weight in relatively large cohorts of CHDs [36,37]. ART pregnancies are also associated with similar complications [3,4,5]. The present results of the increased prevalence of CHDs in ART pregnancies were fully consistent with this background, given the common mesodermal origin of the heart and the placenta.

The retrospective study design was a limitation of our study. Some important variables, such as the ethnicity, socioeconomic status, body mass index, smoker status, acid folic and micronutrients intake and diseases in pregnancy, were not available for many women; thus, we could not analyze them. We distinguished between different types of ART techniques (ICSI vs. IVF) and the origin of gametes (homologous vs. heterologous). However, other variables that could affect the fetal development and pregnancy outcome were not recorded, including different drug protocols of ovarian controlled stimulation, methods of gametes and embryonic micromanipulation, including cryopreservation and PGS/PGD, the transfer of blastocyst vs. cleavage stage embryos and sperm retrieval techniques. Multivariable statistics were not deemed appropriate due to the relatively small sample size of the CHD group.

## 6. Conclusions

Our data showed an increased prevalence of CHDs after ART with a heterogeneous spectrum of anomalies, mainly major defects, the most frequent being Tetralogy of Fallot and HLHS. The figures of our study were consistent with those obtained from the literature. There is a need for further studies intended to examine the contribution of different ART procedures and of different causes of infertility on the development of specific CHDs. The prevalence of CHDs identified in our population of ART pregnancies suggests the opportunity to perform fetal echocardiography due to the potential risk of CHDs associated with this method of conception.

## Figures and Tables

**Table 1:** The characteristics of study population of ART pregnancies.

Characteristics	ART Pregnancies *n* = 1511	%
Maternal age (years)		-
Mean ± SD	37.7 ± 4.71	
range	22–55	
Gestational age at ultrasound (weeks)		-
Mean ± SD	23 ± 4.16	
range	16–37	
Type of exam		
standard cardiac scan (fetal medicine susbspec.)	1124	74.4
fetal echocardiography (fetal cardiologist)	387	25.6
Pregnancy order		
singletons	1242	82.2
multiples	269	17.8
twins	264	98.1 °
triplets	5	1.9 °
dichorionic	230	85.5 °
monochorionic	39	14.5 °
Method of conception		
Homologous ART	1320	87.3
IVF	547	36.2
ICSI	773	51.1
Heterologous ART	191	12.6
Cardiac anatomy		
CHD	29	1.92
non-CHD	1482	98.08

ART—assisted reproductive technology, CHD—congenital heart defects, IVF—in vitro fertilization, ICSI—intracytoplasmic sperm injection, OD—oocyte donation, subspec.—subspecialist. Continuous variables: mean, standard deviation (SD), range. Categorical variables: frequency and percentage. ° The percentage was calculated on the total of multiple pregnancies (*n* = 269).

**Table 2:** Comparison of variables between the two study groups (CHD-ART and non-CHD-ART) in singleton and multiple pregnancies.

**Singleton Pregnancies**			
**VARIABLES**	**CHD ART**	**Non-CHD ART**	***p-*** **Value**
	***n*** **= 23**	***n*** **= 1219**	
Maternal age (years)			
Mean ± SD	39.2 ± 6.37	37.7 ± 4.64	0.129
Range	22–55	22–53	
Gestational age at ultrasound (weeks)			
Mean ± SD	25.8 ± 5.85	23 ± 4.16	0.002 *
Range	28–35	16–37	
Method of conception			
Homologous (total)	15 (65.2)	1081 (88.7)	Homol vs. Heterol 0.003 * Homol IVF vs. ICSI 0.062
Homologous IVF	10/15 (66.6)	441/1081 (40.8)	
Homologous ICSI	5/15 (33.3)	640/1081 (59.2)	
Heterologous	8 (34.8)	138 (11.3)	
**Multiple Pregnancies**			
**VARIABLES**	**CHD ART**	**Non-CHD ART**	***p-*** **Value**
	***n*** **= 5**	***n*** **= 264**	
Maternal age (years)			
Mean ± SD	38.8 ± 5.92	37.7 ± 4.61	0.162
Range	34–47	25–49	
Gestational age at ultrasound (weeks)			
Mean ± SD	29.2 ± 5.51	24.1 ± 4.21	0.147
Range	21–33	16–34	
Method of conception			
Homologous (total)	5 (100)	219 (82.9)	Homol vs. Heterol 0.594 Homol IVF vs. ICSI 0.654
Homologous IVF	2/5 (40)	126/219 (57.5)	
Homologous ICSI	3/5 (60)	93/219 (42.5)	
Heterologous	0	45 (17.1)	

ART—assisted reproductive technology, CHD—congenital heart defects, IVF—in vitro fertilization, ICSI—intracytoplasmic sperm injection; homol—homologous, vs—versus, heterol—heterologous. Continuous variables: mean, standard deviation (SD), range; comparison of variables was performed with Student’s *t*-test. Categorical variables: frequency and percentage; comparison of variables was performed with chi-square test or Fisher exact test as appropriate. For multiple pregnancies the comparison was made on number of pregnancies, though an effective number of fetuses with CHD from twin pregnancies is 6 (in one twin both fetuses were affected). *p*-value < 0.05 was considered significant (*).

**Table 3:** Different types of congenital heart defects according to the type of ART.

Type of CHD	Total *n* CHD	IVF *n*	ICSI *n*	OD *n*
**Major CHD**				
HLHS	4	-	4 (1 twin)	-
ToF/AtrPo+VSD	5	2 (1 twin)	1 twin	2
AVSD	3	1	1	1
TGA	2	2	-	-
DORV	1	1	-	-
Ebstein/Non Ebstein	2	1	1	-
Aortic stenosis	2	1	-	1
Pulmonary stenosis	2	-	-	2
CoA	1			1
Total CHD	22	8	7	7
**Minor CHD**				
VSD	3	2	1 (twin)	
Bicuspid Ao valve	1	-	-	1
PAPVD	1	1	-	-
Persistent LSVC	1	1	-	-
Right Aortic arch	1	1	-	-
Total CHD	7	5	1	1

ART—assisted reproductive technology, CHD—congenital heart defects, IVF—in vitro fertilization, ICSI—intracytoplasmic sperm injection, OD—oocyte donation, *n*—number, HLHS—hypoplastic left heart syndrome, TOF—Tetralogy of Fallot, AtrPo—Pulmonary atresia, VSD—ventricular septal defect, AVSD—atrioventricular septal defect, TGA—transposition of great arteries, DORV—double outlet right ventricle, CoA—coarctation of aorta, ao—aortic, PAPVD—partial anomalous pulmonary vein return, LSVC—left superior vena cava.

## Data Availability

Data are available from the corresponding author upon reasonable request.
